# Natural Killer T Cells in Various Mouse Models of Hepatitis

**DOI:** 10.1155/2021/1782765

**Published:** 2021-01-06

**Authors:** Jun Guan, Gang Wang, Qin Yang, Chao Chen, Jingwen Deng, Xinyu Gu, Haihong Zhu

**Affiliations:** State Key Laboratory for Diagnosis and Treatment of Infectious Diseases, National Clinical Research Center for Infectious Diseases, Collaborative Innovation Center for Diagnosis and Treatment of Infectious Diseases, The First Affiliated Hospital, Zhejiang University School of Medicine, China

## Abstract

Natural killer T (NKT) cells are a key component of innate immunity. Importantly, a growing body of evidence indicates that NKT cells play an integral role in various acute and chronic liver injuries. NKT cells participate in the progression of an injury through the secretion of cytokines, which promote neutrophil infiltration and enhance Fas ligand (FasL) and granzyme-mediated NKT cytotoxic activity. Therefore, examining the role of NKT cells in hepatic disease is critical for a comprehensive understanding of disease pathogenesis and may provide insight into novel approaches for treatment. For more than a century, mouse models that imitate the physiopathological conditions of human disease have served as a critical tool in biological and medical basic research, including studies of liver disease. Here, we review the role of NKT cells in various mouse models of hepatitis.

## 1. Introduction

As the largest parenchymal organ, the liver plays a major role in the metabolism and detoxification of various substances in the body, but also has important immune functions. The liver consists of both parenchymal cells, namely hepatocytes, and nonparenchymal cells. Nonparenchymal cells mainly include liver sinusoidal endothelial cells, Kupffer cells, lymphocytes, cholangio cells, and stellate cells. Lymphocytes can be further divided into T and B cells, natural killer (NK) cells, and natural killer T (NKT) cells. NKT cells are an important part of innate immunity that plays a vital role in liver immunity as the first line of defense against pathogen invasion.

NKT cells constitute a unique subset of mature CD4^+^ T cells that can simultaneously express T cell receptors (TCRs) and NK cell surface proteins (such as CD56 in humans or NK1.1 in mice) [1, 2]. Interestingly, NKT cells account for approximately 30–40% and 5–10% of intrahepatic lymphocytes in mice and humans, respectively [3]. Different from conventional T cells, they are mainly activated by CD1d, a member of the major histocompatibility complex (MHC) class-I-like molecule family, which specifically presents lipid antigens [4]. According to antigen specificities and TCR diversities, CD1d-restricted NKT cells are mainly subdivided into two subsets: type I and type II.

**Figure 1:**
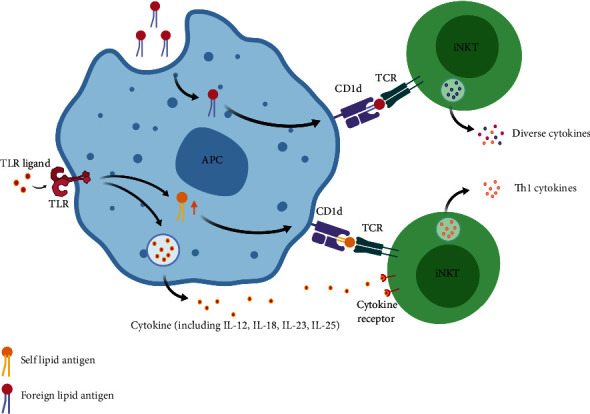
Activation of iNKT cells mainly occurs through one of two signals: T cell receptor (TCR) signaling provided by foreign lipid antigens or proinflammatory cytokine signaling (such as IL-12) produced by PRR-mediated activation of APCs. The TCR signal provided by a self-lipid antigen is also required in physiological conditions. Figure created with BioRender (https://biorender.com). Abbreviations: TCR: T cell receptor; NKT: natural killer T cell; IL: interleukin; APC: antigen-presenting cell; TLR: toll-like receptor; PRR: pattern recognition receptor.

Type I or invariant NKT (iNKT) cells express a semi-invariant TCR (V*α*14-J*α*18 *α* chain with a variable V*β*2, V*β*7, or V*β*8.2 *β* chain in mice or V*α*24-J*α*18 *α* chain with V*β*11 *β* chain in humans) that recognizes self-lipids and microbial lipid antigens [4, 5]. iNKT cells are abundant in the mouse liver (up to 50% of hepatic T cells) but only account for approximately 0.1–1.0% of circulating T lymphocytes and 0.5% of hepatic T cells in humans [6]. *α*-Galactosylceramide (*α*GalCer) is the most effective specific ligand for the activation of iNKT cells [6, 7]. However, as shown in Figure 1, the activation of iNKT cells can occur primarily through two signals: the TCR signal provided by foreign lipid antigens or a proinflammatory cytokine signal that is produced by pattern recognition receptor- (PRR-) mediated activation of antigen-presenting cells (APCs), such as interleukin- (IL-) 12. TCR signalling by self-lipid antigens is also required under physiological conditions [5, 6, 8]. Activated iNKT cells produce various cytokines and simultaneously stimulate dendritic cells (DCs), NK cells, and lymphocytes.

Type II or diverse NKT cells (iiNKT cells) express a diverse array of TCRs that are activated by self-lipids, such as sulfatide and lysophosphatidylcholine (LPC) [6]. In contrast to iNKT cells, iiNKT cells are unable to recognize *α*GalCer [4], and they are the predominant NKT cells in humans. iiNKT cells are more likely to produce Th1 cytokines and can protect against liver injuries by inhibiting the activation of proinflammatory iNKT cells [6, 9]. At present, studies involving iiNKT cells have not been as numerous or detailed as those on iNKT cells.

As a bridge to connect innate and adaptive immunity, NKT cells play an important role in liver diseases by quickly responding to pathogens and secreting inflammatory cytokines to activate downstream immune cells. Therefore, it is necessary to understand the role of NKT cells in hepatitis, which will help to further explore disease pathogenesis and novel treatment strategies. Is it commonly known that liver diseases are a major global burden, especially viral hepatitis (mainly hepatitis B virus (HBV) infection), alcoholic liver disease (ALD), and nonalcoholic fatty liver disease (NAFLD), and these diseases have affected the health of approximately 300 million people in China [10]. Hepatitis can be caused by a variety of factors, including autoimmune reactions, drugs, high-fat diets, alcohol, and hepatitis virus infections. Due to the limited access and supply of human liver samples and manipulation risks, research is normally performed using well-established animal models of hepatitis. Therefore, this review will mainly focus on the indispensable role of NKT cells in various mouse models of hepatitis.

### 1.1. Autoimmune Hepatitis

Autoimmune hepatitis (AIH) is a chronic hepatic inflammatory disease caused by polygenic and multifactorial interactions. The onset can occur at many ages and in both genders worldwide, but the incidence in females is significantly higher compared to males [11]. The diagnosis is based on increased serum transaminase and immunoglobulin G (IgG) levels in addition to the presence of autoantibodies and liver histology indicating interface hepatitis [12]. According to serum autoantibodies, AIH is mainly divided into two types: type 1 (AIH-1) and type 2 (AIH-2). The characteristic autoantibodies of AIH-1 are antinuclear antibodies (ANA) and/or anti-smooth-muscle antibodies (SMA). AIH-2 is less common than AIH-1 and is characterized by a positive test for anti-liver-kidney microsomal antibody type 1 (anti-LKM1), anti-liver-cytosol antibody type 1 antibody (anti-LC1), and/or anti-liver-kidney microsomal antibody type 3 (anti-LKM3) [12, 13].

To date, the hepatitis model induced by intravenous injection of concanavalin A (Con A) is recognized as a well-established T cell-dependent experimental model that closely resembles the pathogenesis of human AIH [14]. It is generally accepted that Con A-induced hepatitis is NKT cell-dependent and NK cell-independent. NKT cells were found to be critical for the induction of AIH, and NKT cell-deficient mice were highly resistant to Con A-induced hepatitis. Additionally, Con A can stimulate NKT cell activation [15], resulting in a rapid early reduction in the number of liver NKT cells [1]. Subsequently, activated NKT cells and T cells, as well as interferon-gamma (IFN-*γ*) and IL-2, mediated the activation of NK cells [16, 17]. NKT cells enhanced cytotoxic activity by upregulating the expression of Fas ligand (FasL) and granzyme B, which can be promoted by IL-4 produced by NKT cells in an autocrine manner [1, 18]. In leukocyte cell-derived chemotaxin-2- (LECT2-) deficient mice, IL-4 and FasL were overexpressed and the liver damage was more severe [19].

Activated iNKT cells have been shown to be involved in liver damage through the secretion of a large number of proinflammatory cytokines, including IFN-*γ*, tumor necrosis factor-alpha (TNF-*α*), osteopontin (OPN), and IL-5. Importantly, IFN-*γ* and TNF-*α* can synergistically promote hepatocyte death. IL-33, an alarm cytokine, is released after hepatocyte injury and can in turn increase the production of IFN-*γ* [20]. IFN-*γ* may also participate in a specific modification that influences the balance of iNKT and iiNKT cell development occurring in the thymus, but the underlying mechanisms require further exploration [21]. iNKT cells mainly play a proinflammatory role, and the activation of iiNKT cells can alleviate inflammation by inducing anergy of iNKT cells [22]. IL-5 was shown to be involved in the maturation and differentiation of eosinophils [23], and IL-15 can ameliorate hepatitis by inhibiting cytokine production (including IL-4 and IL-5) to reduce the infiltration of hepatic eosinophils [24]. The thrombin-cleaved form of OPN enhanced liver damage through the activation of NKT cells and by upregulating the expression of FasL, as well as by stimulating the production of macrophage inflammatory protein- (MIP-) 2 to promote hepatic neutrophil infiltration and activation [25]. Although NK cells also secrete IFN-*γ* and OPN, the severity of hepatitis has been shown to only be related to NKT cells, rather than the function and number of NK cells [25, 26]. However, in glycine N-methyltransferase- (GNMT-) deficient mice that can spontaneously develop nonalcoholic steatohepatitis (NASH), inhibition of NK cells can ameliorate Con A-induced hepatitis [27].

**Figure 2:**
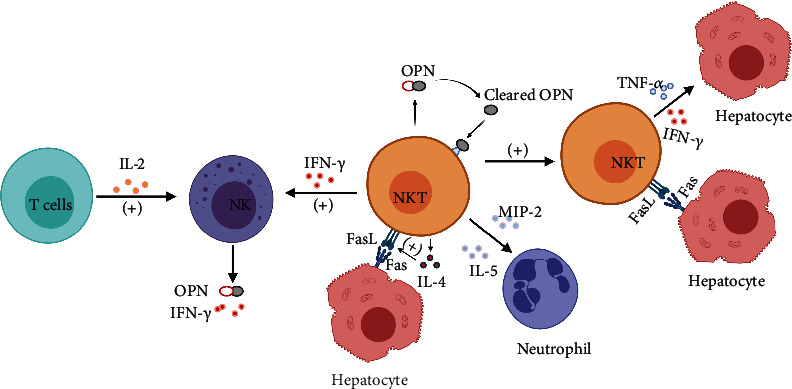
Con A-stimulated NKT cell activation and activated NKT cells secrete IL-4 to promote Fas/FasL-mediated hepatocyte death in an autocrine manner. MIP-2 and IL-5 induce hepatic neutrophil infiltration, and IFN-*γ* and TNF-*α* synergistically kill hepatocytes. Activated NKT cells can also secrete OPN. The thrombin-cleaved form of OPN in turn augments NKT cell activation and FasL expression and secretion of MIP-2 to enhance liver damage. NKT cell-secreted IFN-*γ* and T cell-secreted IL-2 further trigger the activation of NK cells. Activated NK cells cosecrete OPN and IFN-*γ* to help NKT cells exert a proinflammatory effect. Figure created with BioRender (https://biorender.com). Abbreviations: Con A: concanavalin A; (+): activate; NK: natural killer cell; NKT: natural killer T cell; IL: interleukin; OPN: osteopontin; IFN-*γ*: interferon-gamma; MIP-2: macrophage inflammatory protein-2; TNF-*α*: tumor necrosis factor-alpha; FasL: Fas ligand.

Activated NKT cells have also been shown to promote IL-6-mediated liver regeneration after partial hepatectomy [28]. Treatment with retinoic acid (RA) attenuated Con A-induced hepatitis by directly altering retinoic acid receptor alpha (RAR-*α*) and mitogen-activated protein kinase- (MAPK-) related signaling molecules, which reduced IFN-*γ* and IL-4 [29]. Treatment with prednisolone also ameliorated Con A-induced liver damage, but the exact mechanism remains unclear [30]. In addition, regulation of DC-mediated NKT cell activation by the gut microbiota can affect liver inflammation, which may elicit new ideas for clinical treatment [31].  Figure 2 summarizes the roles of NKT cells in AIH.

### 1.2. Drug-Induced Hepatitis

The liver is an important organ of drug metabolism, and it is more susceptible to damage due to the toxicity of the drugs themselves or their metabolites. Drug-induced liver injury, especially acetaminophen (*N*-acetyl-p-aminophenol, APAP) overdose, is the most common cause of acute liver failure in Western countries [32–34]. *N*-acetyl-p-benzoquinone-imine (NAPQI) is a toxic metabolite of APAP that can combine with glutathione for detoxification. When glutathione is depleted, NAPQI binds to cellular proteins, especially mitochondrial proteins, leading to mitochondrial oxidative stress and dysfunction and eventually leading to hepatocyte necrosis [35–37].

A mouse model whereby APAP is administered via intraperitoneal injection can mimic the mechanism of a patient-like injury and is the most widely used model for studying the pathogenesis of drug-induced hepatitis [38, 39]. The roles of NKT cells in APAP-mediated drug-induced hepatitis remain controversial. One study found that compared to wild-type (WT) mice, the severity of liver injury in NK cell-deficient or NKT cell-deficient mice was similar [40]. Only when NK and NKT cells were simultaneously defective were the symptoms of hepatitis significantly improved, which may be related to the functional overlap between these two cell types. After APAP administration, NK and NKT cells secreted a large amount of IFN-*γ*, upregulated the expression of FasL on innate immune cells, regulated the secretion of various chemokines, and promoted the infiltration of hepatic inflammatory cells, including neutrophils, macrophages, NK cells, NKT cells, and T cells [40]. Masson et al. found that liver injury was not alleviated in NK- and NKT cell-deficient mice when saline was used as a solvent for APAP as the former study described; this phenomenon could only occur when DMSO was used as the solvent for APAP. Although DMSO does not cause liver damage directly, it can be involved in liver injury by increasing the number of NKT cells and inducing the expression of cytotoxic effector molecules (granzyme B and IFN-*γ*) by NK and NKT cells [39].

Another study has shown that NKT cell-deficient mice were more susceptible to APAP-mediated drug-induced hepatitis [38]. The administration of APAP markedly upregulated ketone bodies and cytochrome P450 2E1 (CYP2E1) in NKT cell-deficient mice, and the APAP-protein adduct formed through the binding of NAPQI (APAP metabolite) to hepatocellular proteins increased. This APAP-protein adduct formation induced mitochondrial oxidative stress and dysfunction, resulting in an increased susceptibility of the NKT cell-deficient mice to APAP-induced hepatitis. Interestingly, compared to WT mice, there was no marked difference in cytokine levels. The difference was primarily found in ketone bodies and CYP2E1, suggesting that NKT cells may be closely related to metabolism [38]. In addition, granzyme B-deficient mice can experience aggravated liver damage through an increased number and greater activation of NK and NKT cells, as well as FasL expression [32].

### 1.3. Nonalcoholic Steatohepatitis

NAFLD is the most commonly diagnosed cause of chronic liver disease in Western countries and is characterized by hepatic parenchymal cell steatosis and fat accumulation without a history of excessive drinking [41, 42]. The disease spectrum ranges from simple steatosis to nonalcoholic steatohepatitis (NASH), the latter of which can develop into cirrhosis and hepatocellular carcinoma [9]. NASH is strongly correlated with metabolic syndrome and visceral adiposity [43]. In addition to steatosis, lobular inflammation and ballooning hepatocellular injury are pathological features of NASH [44].

There are three main animal models of NASH, including a dietary model, genetic model, and chemically induced model [41, 45]. Different animal models have their own advantages and disadvantages. The ideal animal model should be able to simulate all the characteristics of the human condition and reflect the multifactorial nature of the mechanism of disease pathogenesis, but currently, there is no animal model that can perfectly simulate the conditions present in NASH patients. Some researchers believe that the diet-induced obesity model, such as the FFC diet (high fat, fructose, and cholesterol), is best [41], and NKT cells participate in the pathogenesis of diet-induced NASH. Consumption of a high-fat diet (HFD) increased the secretion of IL-12, a cytokine that inhibits the activity of NKT cells. IL-12 reduced the number of NKT cells by mediating apoptosis of hepatic NKT cells, but the remaining cells were able to produce more proinflammatory cytokines, such as IFN-*γ* and TNF-*α* [46]. In choline-deficient L-amino acid-defined (CDAA) diet-induced NASH, the expression of TCR and NK1.1 on iNKT cells was downregulated, and the secreted cytokine profile changed from IL-17 to IFN-*γ* and IL-4. iNKT cells also contributed to hepatic infiltration of both CD8^+^ T cells and Kupffer cells (KCs) [47]. Intraperitoneal injection of *α*-GalCer effectively activated NKT cells, increased the frequency of iNKT cells (mainly iNKT2 cells, a subset of iNKT cells that express high levels of promyelocytic leukaemia zinc finger (PLZF) and GATA3), induced IL-4 secretion, and eventually improved liver steatosis [48]. In addition, probiotics can also improve liver NKT cell depletion as well as alleviate insulin resistance and hepatic steatosis [49].

NKT cells have been shown to be trapped in NASH formation and progression of NASH-associated fibrosis. In the early stage of methionine choline-deficient (MCD) diet-induced NASH, IL-15 and chemokine (C-X-C motif) receptor 6 (CXCR6) mediated hepatic NKT cell accumulation. NKT cells produced a large quantity of IL-4, IFN-*γ*, and OPN; promoted macrophage infiltration; and enhanced the inflammatory response [50, 51]. During MCD diet-induced NASH-associated fibrosis, the Hedgehog (Hh) signaling pathway was activated, which in turn promoted the production of chemokine (C-X-C motif) ligand 16 (CXCL16) and mediated the recruitment of NKT cells to the liver. Liver NKT cells produced and responded to the Hh ligand, Sonic Hedgehog (SHh), which not only directly stimulated the transformation of hepatic stellate cells (HSCs) into myofibroblasts but also indirectly triggered myofibroblast activation by increasing the production of the profibrotic cytokines (IL-4 and IL-13) from NKT cells. Importantly, NKT cell-deficient mice were protected from MCD and CDAA diet-associated hepatic steatosis and fibrosis [47, 52].

However, an opposing view has been suggested whereby NKT cells play a protective role in the pathogenesis of NASH-associated fibrosis. iNKT cells can inhibit HFD-mediated hepatitis, and iNKT cell-deficient mice were prone to the development of steatohepatitis and liver fibrosis [53]. This difference in effect may be related to different genetic backgrounds and dietary habits of the mice. Different dietary habits can lead to a discrepancy in immune cell activation and liver microecology.

### 1.4. Alcoholic Steatohepatitis

ALD is the leading cause of alcohol-related deaths, and the disease spectrum ranges from simple steatosis to more severe stages, including steatohepatitis with or without fibrosis, cirrhosis, and hepatocellular cancer [54]. In China, a dramatic increase in alcohol consumption has made ALD a leading cause of end-stage liver disease, second only to viral hepatitis [55]. Many factors are involved in the pathogenesis of ALD, including alcohol itself and its metabolites, acetaldehyde-mediated hepatocellular injury, the immune system response to injury, changes in intestinal permeability, and microecological disorders [56].

The mouse models of alcoholic liver injury include acute oral gavage, *ad libitum* access to alcohol in the drinking water, intragastric infusion (Tsukamoto-French model), chronic Lieber-DeCarli diet ethanol feeding, and the chronic-binge ethanol model (NIAAA model). Unfortunately, none of these models can represent the complete pathogenesis of ALD in human patients [57, 58]. The following studies center mainly around the chronic-plus-single-binge ethanol consumption mouse model that is easy to perform and has marked steatosis. Chronic-plus-binge ethanol feeding has been shown to induce alcoholic steatohepatitis and not only does the liver damage depends on iNKT cells, the iNKT cells themselves can aggravate the hepatitis. ALD has been shown to be alleviated when all-trans retinoic acid (atRA) signals through the retinoic acid receptor gamma (RAR*γ*) to directly inhibit iNKT cells or when sulfatide activation of iiNKT cells indirectly inhibits iNKT cells [39].

Ethanol-mediated increased expression of CD1d on intestinal epithelial cells and hepatocytes has been shown to lead to the migration of iNKT cells from mesenteric lymph nodes to the liver and subsequently activate liver iNKT cells [59, 60]. KC-derived NLRP3 inflammasome activation and NLRP3-mediated IL-1*β* secretion were shown to be required for hepatic iNKT cell accumulation and activation [61]. Liver steatosis was accompanied by an increased number, frequency, and activation of iNKT cells. iNKT cells promoted neutrophil infiltration and hepatic inflammation by upregulating the expression of cytokines and chemokines relevant to neutrophil infiltration, including OPN, IL-4, IL-6, MIP-1*α*, MIP-2, and TNF-*α* [61, 62]. TNF-*α* exerted a proinflammatory effect in an E-selectin-dependent manner, whereas IL-4 was not essential [61].

Activated iNKT cells also can inhibit NK cell frequency, number, and function mediated by IL-10. *In vitro*, NK cells and secreted IFN-*γ* have been shown to protect the liver from steatosis by directly reducing hepatocyte lipogenesis [63]. Long-term ethanol feeding can antagonize this protective effect in the progression of alcoholic steatohepatitis-associated fibrosis. Chronic ethanol consumption was shown to accelerate the progression of fibrosis by (1) elevating the secretion of transforming growth factor-beta 1 (TGF-*β*1) by HSCs, which downregulated NKG2D and TNF-related apoptosis-inducing ligand (TRAIL) to result in a reduced capacity of NK cells to kill HSCs, and (2) impairing the antifibrotic effects of IFN-*γ* by interrupting IFN-*γ*/signal transducer and activator of transcription 1 (STAT1) signaling in HSCs [64].

**Figure 3:**
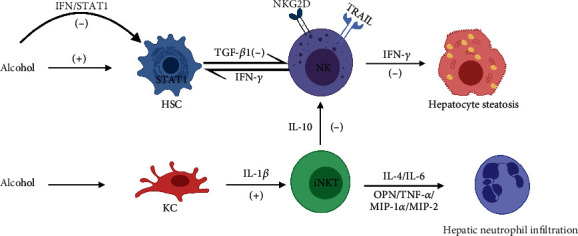
Alcohol stimulates KC to secrete IL-1*β*, which mediates the accumulation and activation of liver iNKT cells. iNKT cells promote neutrophil infiltration and liver inflammation by upregulating the expression of OPN, IL-4, IL-6, MIP-1*α*, MIP-2, and TNF-*α*. Activated iNKT cells also secrete IL-10 to inhibit the number and function of NK cells. *In vitro*, NK cells and secreted IFN-*γ* can protect the liver from steatosis. Chronic ethanol consumption stimulates HSCs to secrete TGF-*β*1 to downregulate NKG2D and TRAIL expression on NK cells, leading to an impaired ability to kill HSCs and antagonizing the IFN-*γ* antifibrosis effect. Figure created with BioRender (https://biorender.com). Abbreviations: (+): activate; (-): inhibit; NK: natural killer cell; NKT: natural killer T cell; IL: interleukin; OPN: osteopontin; IFN-*γ*: interferon-gamma; MIP: macrophage inflammatory protein; TNF-*α*: tumor necrosis factor-*α*; TGF-*β*1: transforming growth factor-*β*1; HSC: hepatic stellate cell; KC: Kupffer cell; TRAIL: TNF-related apoptosis-inducing ligand; HSC: hepatic stellate cells.

However, the role of IFN-*γ* in ALD remains controversial. IFN-*γ* is mainly secreted by NKT and NK cells. One study has shown that there was no significant change in the levels of IFN-*γ* produced by iNKT cells or in serum alanine aminotransferase (ALT) levels between IFN-*γ*-deficient mice and WT mice [62]. These results suggested that IFN-*γ* may not be involved in liver damage, although another study suggested that IFN-*γ* secreted by NK cells had protective effects, and the liver damage and steatosis in IFN-*γ*-deficient mice were more severe compared to WT mice [63]. The above two studies regarding IFN-*γ* differed due to the cellular source of IFN-*γ*, and additional studies should be performed and comprehensively analyzed to determine the precise mechanism for this observed difference. The roles of NKT cells in alcoholic steatohepatitis have been summarized in Figure 3.

### 1.5. Hepatitis B

Viral hepatitis is a serious global infectious disease caused by a variety of viruses, including hepatitis A, B, C, D, and E viruses that target the liver as the primary source of infection and characteristically present with inflammatory liver damage and abnormal liver function as the main pathological and clinical manifestations. Among them, HBV infection is the most common chronic hepatitis viral infection that can develop into cirrhosis and even hepatocellular carcinoma in some severe cases. The number of chronic HBV carriers worldwide has risen to exceed 350 million people, and it has become an urgent global public health problem [65]. There are very few studies that have been performed in mouse models of HAV, HCV, HDV, and HEV infections that have also investigated the role of NKT cells. Therefore, for the purpose of this review, the focus will be on the role of NKT cells in hepatitis B.

Using the HBV transgenic mouse model can accurately represent the health carrier status of a human with an HBV infection [66]. Although the transgenic mouse model is currently the most widely used animal model for studying HBV, it also has limitations due to the inability to study human HBV natural infection and immune clearance mechanisms.

Results from this mouse model have revealed that after HBV infection of hepatocytes, the self-phospholipid antigen phosphatidylethanolamine (PE) and secretory phospholipid (sPLA2) enzymes were increased. sPLA2 induced the conversion of PE to lysoPE. CD1d on hepatocytes presented lysoPE, which was able to activate iiNKT cells. Additionally, DCs produced IL-12 to contribute to iNKT cell activation [67]. Also, NKG2D may act as a costimulatory molecule, interacting with other ligands to mediate NKT cell activation [68]. Activated NKT cells were shown to contribute to the induction and proliferation of HBV-specific cytotoxic T lymphocytes (CTLs), as well as the activation of NK, T, and B cells, and helped to establish innate and adaptive immunity against HBV [69, 70].

In HBV transgenic mice with acute hepatitis, NKT cells were essential for the induction of acute hepatitis, which was accompanied by increased NKT cell number and activation [71]. In chronic hepatitis, the frequency of NKT cells was reduced and the ability to eliminate the virus was impaired. NKT cells upregulated programmed cell death protein 1 (PD-1) and downregulated CD28, and importantly, activating the CD28/CD80 pathway or blocking PD1/PDL1 signaling can effectively modulate the function to inhibit HBV replication [72]. In HBV patients, NKT cells have been shown to be decreased, but the number can gradually return to normal following antiviral therapy [73].

**Figure 4:**
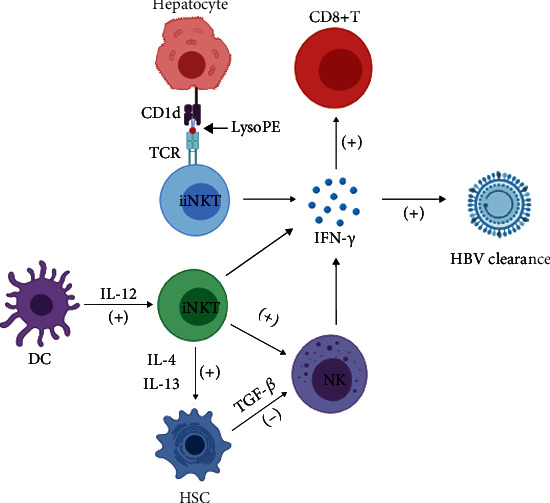
CD1d on hepatocytes present lysoPE to activate iiNKT cells. DCs secrete IL-12 to trigger iNKT cell activation. Activated NKT cells contribute to NK cell activation. Activated NKT and NK cells secrete IFN-*γ* to clear HBV. IFN-*γ* also enhances CD8^+^ T cell immunity. Activated hepatic iNKT cells secrete IL-4 and IL-13 to activate HSCs and enhance liver fibrosis. HSCs can inhibit the antifibrotic effect of NK cells by secreting TGF-*β*. Figure created with BioRender (https://biorender.com). Abbreviations: (+): activate; (-): inhibit; PE: phosphatidylethanolamine; TCR: T cell antigen receptor; NK: natural killer cell; NKT: natural killer T cell; IFN-*γ*: interferon-*γ*; HBV: hepatitis B virus; DC: dendritic cell; IL: interleukin; TGF-*β*: transforming growth factor-*β*; HSC: hepatic stellate cell.

Activated NKT cells secrete Th1- and Th2-type cytokines, such as IFN-*γ* and IFN-*α*/*β*, both of which recruit inflammatory cells into the liver to enhance liver damage, and contribute to viral clearance [74]. Moreover, IFN-*γ* can also inhibit hepatocyte regeneration by negatively regulating the cell cycle [75]. In patients with HBV-associated liver fibrosis, the frequency and number of circulating iNKT cells were reduced, but their activation was enhanced. Activated iNKT cells were shown to migrate to the liver and secrete IL-4 and IL-13 to activate HSCs that resulted in enhanced liver fibrosis [76, 77]. Additionally, HSCs secrete TGF-*β* to inhibit the antifibrotic activity of NK cells and accelerate the progression of liver fibrosis [78]. The roles of NKT cells in hepatitis B and associated liver fibrosis have been illustrated in Figure 4.

## 2. Conclusion

Although the major subset of NKT cells in the livers of mice and humans is different, since iNKT cells are predominant in murine livers whereas iiNKT cells are more common in human livers, mouse models of hepatitis remain one of the most important animal models for the study of human liver diseases [79, 80]. NKT cells represent a unique subset of cells sharing certain characteristics of both T and NK cells. The liver is an important organ participating in the immune regulation of the entire body. Compared to other organs, the liver has the most abundant number of NKT cells and the two subtypes of NKT cells have opposing functions. Thus, NKT cells have the potential to regulate liver immunity during hepatitis. Despite the fact that the pathogenesis of hepatitis can be induced by a variety of factors, it has been confirmed that NKT cells and their secreted cytokines play irreplaceable roles in the various forms of hepatitis. With related research that is currently being undertaken, there is a growing body of evidence that supports novel and emerging therapeutic targets for the treatment of the many types of hepatitis. Meanwhile, the mouse model is expected to greatly support these new experimental findings regarding the mechanisms underlying human liver disease initiation and progression.

## References

[1] Takeda K., Hayakawa Y., Van Kaer L., Matsuda H., Yagita H., Okumura K. (2000). Critical contribution of liver natural killer T cells to a murine model of hepatitis. *Proceedings of the National Academy of Sciences of the United States of America*.

[2] Bendelac A., Killeen N., Littman D., Schwartz R. (1994). A subset of CD4+ thymocytes selected by MHC class I molecules. *Science*.

[3] Gao B., Jeong W. I., Tian Z. (2008). Liver: an organ with predominant innate immunity. *Hepatology*.

[4] Rossjohn J., Pellicci D. G., Patel O., Gapin L., Godfrey D. I. (2012). Recognition of CD1d-restricted antigens by natural killer T cells. *Nature Reviews. Immunology*.

[5] Brennan P. J., Brigl M., Brenner M. B. (2013). Invariant natural killer T cells: an innate activation scheme linked to diverse effector functions. *Nature Reviews. Immunology*.

[6] Bandyopadhyay K., Marrero I., Kumar V. (2016). NKT cell subsets as key participants in liver physiology and pathology. *Cellular & Molecular Immunology*.

[7] Kawano T., Cui J., Koezuka Y. (1997). CD1d-restricted and TCR-mediated activation of valpha14 NKT cells by glycosylceramides. *Science*.

[8] Kitamura H., Iwakabe K., Yahata T. (1999). The natural killer T (NKT) cell ligand alpha-galactosylceramide demonstrates its immunopotentiating effect by inducing interleukin (IL)-12 production by dendritic cells and IL-12 receptor expression on NKT cells. *The Journal of Experimental Medicine*.

[9] Tajiri K., Shimizu Y., Tsuneyama K., Sugiyama T. (2009). Role of liver-infiltrating CD3+CD56+ natural killer T cells in the pathogenesis of nonalcoholic fatty liver disease. *European Journal of Gastroenterology & Hepatology*.

[10] Wang F. S., Fan J. G., Zhang Z., Gao B., Wang H. Y. (2014). The global burden of liver disease: the major impact of China. *Hepatology*.

[11] Heneghan M. A., Yeoman A. D., Verma S., Smith A. D., Longhi M. S. (2013). Autoimmune hepatitis. *Lancet*.

[12] Mieli-Vergani G., Vergani D., Czaja A. J. (2018). Autoimmune hepatitis. *Nature Reviews Disease Primers*.

[13] Manns M. P., Lohse A. W., Vergani D. (2015). Autoimmune hepatitis--update 2015. *Journal of Hepatology*.

[14] Wang H. X., Liu M., Weng S. Y. (2012). Immune mechanisms of Concanavalin A model of autoimmune hepatitis. *World Journal of Gastroenterology*.

[15] Wei Y., Zeng B., Chen J. (2016). Enterogenous bacterial glycolipids are required for the generation of natural killer T cells mediated liver injury. *Scientific Reports*.

[16] Miyagi T., Takehara T., Tatsumi T. (2004). Concanavalin a injection activates intrahepatic innate immune cells to provoke an antitumor effect in murine liver. *Hepatology*.

[17] Huang J., Yuan Q., Zhu H. (2017). IL-17C/IL-17RE augments T cell function in autoimmune hepatitis. *Journal of Immunology*.

[18] Kaneko Y., Harada M., Kawano T. (2000). Augmentation of Valpha14 NKT cell-mediated cytotoxicity by interleukin 4 in an autocrine mechanism resulting in the development of concanavalin A-induced hepatitis. *The Journal of Experimental Medicine*.

[19] Saito T., Okumura A., Watanabe H. (2004). Increase in hepatic NKT cells in leukocyte cell-derived chemotaxin 2-deficient mice contributes to severe concanavalin A-induced hepatitis. *Journal of Immunology*.

[20] Chen J., Duan L., Xiong A. (2012). Blockade of IL-33 ameliorates Con A-induced hepatic injury by reducing NKT cell activation and IFN-*γ* production in mice. *Journal of Molecular Medicine (Berlin, Germany)*.

[21] Hashimoto M., Hiwatashi K., Ichiyama K. (2011). SOCS1 regulates type I/type II NKT cell balance by regulating IFNgamma signaling. *International Immunology*.

[22] Halder R. C., Aguilera C., Maricic I., Kumar V. (2007). Type II NKT cell-mediated anergy induction in type I NKT cells prevents inflammatory liver disease. *The Journal of Clinical Investigation*.

[23] Louis H., Le Moine A., Flamand V. (2002). Critical role of interleukin 5 and eosinophils in concanavalin A-induced hepatitis in mice. *Gastroenterology*.

[24] Li B., Sun R., Wei H., Gao B., Tian Z. (2006). Interleukin-15 prevents concanavalin A-induced liver injury in mice via NKT cell-dependent mechanism. *Hepatology*.

[25] Diao H., Kon S., Iwabuchi K. (2004). Osteopontin as a mediator of NKT cell function in T cell-mediated liver diseases. *Immunity*.

[26] Filliol A., Piquet-Pellorce C., Dion S. (2017). PARP2 deficiency affects invariant-NKT-cell maturation and protects mice from concanavalin A-induced liver injury. *American Journal of Physiology. Gastrointestinal and Liver Physiology*.

[27] Gomez-Santos L., Luka Z., Wagner C. (2012). Inhibition of natural killer cells protects the liver against acute injury in the absence of glycine N-methyltransferase. *Hepatology*.

[28] Ya’acov A. B., Meir H., Zolotaryova L., Ilan Y., Shteyer E. (2017). Impaired liver regeneration is associated with reduced cyclin B1 in natural killer T cell-deficient mice. *BMC Gastroenterology*.

[29] Maricic I., Sheng H., Marrero I. (2015). Inhibition of type I natural killer T cells by retinoids or following sulfatide-mediated activation of type II natural killer T cells attenuates alcoholic liver disease in mice. *Hepatology*.

[30] Kwon H. J., Won Y. S., Park O., Feng D., Gao B. (2014). Opposing effects of prednisolone treatment on T/NKT cell- and hepatotoxin-mediated hepatitis in mice. *Hepatology*.

[31] Chen J., Wei Y., He J. (2015). Natural killer T cells play a necessary role in modulating of immune- mediated liver injury by gut microbiota. *Scientific Reports*.

[32] Getachew Y., Cusimano F. A., James L. P., Thiele D. L. (2014). The role of intrahepatic CD3+/CD4-/CD8- double negative T (DN T) cells in enhanced acetaminophen toxicity. *Toxicology and Applied Pharmacology*.

[33] Downs I., Aw T. Y., Liu J., Adegboyega P., Ajuebor M. N. (2012). V*α*14iNKT cell deficiency prevents acetaminophen-induced acute liver failure by enhancing hepatic glutathione and altering APAP metabolism. *Biochemical and Biophysical Research Communications*.

[34] Katarey D., Verma S. (2016). Drug-induced liver injury. *Clinical Medicine (London, England)*.

[35] Lee W. M. (2017). Acetaminophen (APAP) hepatotoxicity-isn't it time for APAP to go away?. *Journal of Hepatology*.

[36] Dahlin D. C., Miwa G. T., Lu A. Y., Nelson S. D. (1984). N-acetyl-p-benzoquinone imine: a cytochrome P-450-mediated oxidation product of acetaminophen. *Proceedings of the National Academy of Sciences of the United States of America*.

[37] Yan M., Huo Y., Yin S., Hu H. (2018). Mechanisms of acetaminophen-induced liver injury and its implications for therapeutic interventions. *Redox Biology*.

[38] Martin-Murphy B. V., Kominsky D. J., Orlicky D. J., Donohue T. M., Ju C. (2013). Increased susceptibility of natural killer T-cell-deficient mice to acetaminophen-induced liver injury. *Hepatology*.

[39] Masson M. J., Carpenter L. D., Graf M. L., Pohl L. R. (2008). Pathogenic role of natural killer T and natural killer cells in acetaminophen-induced liver injury in mice is dependent on the presence of dimethyl sulfoxide. *Hepatology*.

[40] Liu Z. X., Govindarajan S., Kaplowitz N. (2004). Innate immune system plays a critical role in determining the progression and severity of acetaminophen hepatotoxicity. *Gastroenterology*.

[41] Ibrahim S. H., Hirsova P., Malhi H., Gores G. J. (2016). Animal models of nonalcoholic steatohepatitis: eat, delete, and inflame. *Digestive Diseases and Sciences*.

[42] Byrne C. D., Targher G. (2015). NAFLD: a multisystem disease. *Journal of Hepatology*.

[43] Diehl A. M., Day C. (2017). Cause, pathogenesis, and treatment of nonalcoholic steatohepatitis. *The New England Journal of Medicine*.

[44] Bedossa P. (2017). Pathology of non-alcoholic fatty liver disease. *Liver International*.

[45] Hansen H. H., Feigh M., Veidal S. S., Rigbolt K. T., Vrang N., Fosgerau K. (2017). Mouse models of nonalcoholic steatohepatitis in preclinical drug development. *Drug Discovery Today*.

[46] Li Z., Soloski M. J., Diehl A. M. (2005). Dietary factors alter hepatic innate immune system in mice with nonalcoholic fatty liver disease. *Hepatology*.

[47] Maricic I., Marrero I., Eguchi A. (2018). Differential activation of hepatic invariant NKT cell subsets plays a key role in progression of nonalcoholic steatohepatitis. *Journal of Immunology*.

[48] Chen D., Gao X., Wang J. (2019). Activation of hepatic iNKT2 cells by *α*-GalCer ameliorates hepatic steatosis induced by high-fat diet in C57BL/6J mice. *International Immunopharmacology*.

[49] Ma X., Hua J., Li Z. (2008). Probiotics improve high fat diet-induced hepatic steatosis and insulin resistance by increasing hepatic NKT cells. *Journal of Hepatology*.

[50] Wehr A., Baeck C., Heymann F. (2013). Chemokine receptor CXCR6-dependent hepatic NK T cell accumulation promotes inflammation and liver fibrosis. *Journal of Immunology*.

[51] Locatelli I., Sutti S., Vacchiano M., Bozzola C., Albano E. (2013). NF-*κ*B1 deficiency stimulates the progression of non-alcoholic steatohepatitis (NASH) in mice by promoting NKT-cell-mediated responses. *Clinical Science (London, England)*.

[52] Syn W.-K., Oo Y. H., Pereira T. A. (2010). Accumulation of natural killer T cells in progressive nonalcoholic fatty liver disease. *Hepatology*.

[53] Miyagi T., Takehara T., Uemura A. (2010). Absence of invariant natural killer T cells deteriorates liver inflammation and fibrosis in mice fed high-fat diet. *Journal of Gastroenterology*.

[54] Marroni C. A., Alfeu de Medeiros Fleck S. A. F., Galant L. H. (2018). Liver transplantation and alcoholic liver disease: history, controversies, and considerations. *World Journal of Gastroenterology*.

[55] You Ming L. I., FAN J. G., WANG B. Y. (2011). Guidelines for the diagnosis and management of alcoholic liver disease: update 2010: (published in Chinese on Chinese Journal of Hepatology 2010; 18: 167-170). *Journal of Digestive Diseases*.

[56] Dunn W., Shah V. H. (2016). Pathogenesis of alcoholic liver disease. *Clinics in Liver Disease*.

[57] Williams J. A., Manley S., Ding W. X. (2014). New advances in molecular mechanisms and emerging therapeutic targets in alcoholic liver diseases. *World Journal of Gastroenterology*.

[58] Bertola A., Mathews S., Ki S. H., Wang H., Gao B. (2013). Mouse model of chronic and binge ethanol feeding (the NIAAA model). *Nature Protocols*.

[59] Chelakkot-Govindalayathil A. L., Mifuji-Moroka R., D'Alessandro-Gabazza C. N. (2015). Protein S exacerbates alcoholic hepatitis by stimulating liver natural killer T cells. *Journal of Thrombosis and Haemostasis*.

[60] Lee K. C., Chen P., Maricic I. (2019). Intestinal iNKT cells migrate to liver and contribute to hepatocyte apoptosis during alcoholic liver disease. *American Journal of Physiology. Gastrointestinal and Liver Physiology*.

[61] Cui K., Yan G., Xu C. (2015). Invariant NKT cells promote alcohol-induced steatohepatitis through interleukin-1*β* in mice. *Journal of Hepatology*.

[62] Mathews S., Feng D., Maricic I., Ju C., Kumar V., Gao B. (2016). Invariant natural killer T cells contribute to chronic-plus-binge ethanol-mediated liver injury by promoting hepatic neutrophil infiltration. *Cellular & Molecular Immunology*.

[63] Cui K., Yan G., Zheng X. (2017). Suppression of natural killer cell activity by regulatory NKT10 cells aggravates alcoholic hepatosteatosis. *Frontiers in Immunology*.

[64] Jeong W. I., Park O., Gao B. (2008). Abrogation of the Antifibrotic Effects of Natural Killer Cells/Interferon-*γ* Contributes to Alcohol Acceleration of Liver Fibrosis. *Gastroenterology*.

[65] Trepo C., Chan H. L., Lok A. (2014). Hepatitis B virus infection. *Lancet*.

[66] Chisari F. V., Pinkert C. A., Milich D. R. (1985). A transgenic mouse model of the chronic hepatitis B surface antigen carrier state. *Science*.

[67] Godfrey D. I., Uldrich A. P., Baxter A. G. (2012). NKT cells--an early warning system for HBV infection. *Nature Medicine*.

[68] Vilarinho S., Ogasawara K., Nishimura S., Lanier L. L., Baron J. L. (2007). Blockade of NKG2D on NKT cells prevents hepatitis and the acute immune response to hepatitis B virus. *Proceedings of the National Academy of Sciences of the United States of America*.

[69] Zeissig S., Murata K., Sweet L. (2012). Hepatitis B virus-induced lipid alterations contribute to natural killer T cell-dependent protective immunity. *Nature Medicine*.

[70] Ito H., Ando K., Ishikawa T. (2008). Role of Valpha14+ NKT cells in the development of hepatitis B virus-specific CTL: activation of Valpha14+ NKT cells promotes the breakage of CTL tolerance. *International Immunology*.

[71] Baron J. L., Gardiner L., Nishimura S., Shinkai K., Locksley R., Ganem D. (2002). Activation of a nonclassical NKT cell subset in a transgenic mouse model of hepatitis B virus infection. *Immunity*.

[72] Wang X. F., Lei Y., Chen M., Chen C. B., Ren H., Shi T. D. (2013). PD-1/PDL1 and CD28/CD80 pathways modulate natural killer T cell function to inhibit hepatitis B virus replication. *Journal of Viral Hepatitis*.

[73] Li J., Han Y., Jin K. (2011). Dynamic changes of cytotoxic T lymphocytes (CTLs), natural killer (NK) cells, and natural killer T (NKT) cells in patients with acute hepatitis B infection. *Virology Journal*.

[74] Kakimi K., Guidotti L. G., Koezuka Y., Chisari F. V. (2000). Natural killer T cell activation inhibits hepatitis B virus replication in vivo. *The Journal of Experimental Medicine*.

[75] Dong Z., Zhang J., Sun R., Wei H., Tian Z. (2007). Impairment of liver regeneration correlates with activated hepatic NKT cells in HBV transgenic mice. *Hepatology*.

[76] Wei X., Qian J., Yao W. (2019). Hyperactivated peripheral invariant natural killer T cells correlate with the progression of HBV-relative liver cirrhosis. *Scandinavian Journal of Immunology*.

[77] Jin Z., Sun R., Wei H., Gao X., Chen Y., Tian Z. (2011). Accelerated liver fibrosis in hepatitis B virus transgenic mice: involvement of natural killer T cells. *Hepatology*.

[78] Shi J., Zhao J., Zhang X. (2017). Activated hepatic stellate cells impair NK cell anti-fibrosis capacity through a TGF-*β*-dependent emperipolesis in HBV cirrhotic patients. *Scientific Reports*.

[79] Berzins S. P., Smyth M. J., Baxter A. G. (2011). Presumed guilty: natural killer T cell defects and human disease. *Nature Reviews. Immunology*.

[80] Emoto M., Kaufmann S. H. E. (2003). Liver NKT cells: an account of heterogeneity. *Trends in Immunology*.

